# The effect of stress on delay discounting in female patients with early-onset bulimia nervosa and alcohol use disorder: functional magnetic resonance imaging study

**DOI:** 10.1192/bjo.2025.10821

**Published:** 2025-09-12

**Authors:** Nicolas Leenaerts, Jenny Ceccarini, Martin Weygandt, Stefan Sunaert, Elske Vrieze

**Affiliations:** Department of Neurosciences, Research Group Psychiatry, KU Leuven, Leuven Brain Institute, Leuven, Belgium; Department of Neurosciences, Mind-Body Research, Research Group Psychiatry, KU Leuven, Leuven, Belgium; Department of Nuclear Medicine and Molecular Imaging, Research Nuclear Medicine & Molecular Imaging, KU Leuven, Leuven Brain Institute, Leuven, Belgium; Experimental and Clinical Research Center, a Cooperation between the Max Delbrück Center for Molecular Medicine in the Helmholtz Association and Charité Universitätsmedizin Berlin, Berlin, Germany; Charité—Universitätsmedizin Berlin, Corporate Member of Freie Universität Berlin and Humboldt-Universität zu Berlin, Experimental and Clinical Research Center, Berlin, Germany; Max Delbrück Center for Molecular Medicine in the Helmholtz Association (MDC), Berlin, Germany; Translational MRI, Department of Imaging and Pathology, Biomedical Sciences Group, KU Leuven, Leuven, Belgium

**Keywords:** Delay discounting, stress, bulimia nervosa, alcohol use disorder, fMRI

## Abstract

**Aims:**

Stress could increase delay discounting in subjects with bulimia nervosa and alcohol use disorder (AUD), meaning that the short-term benefits of coping through eating or drinking outweigh the long-term negative consequences. Therefore, this study explores differences in delay discounting between patients and healthy controls, the impact of stress on food and alcohol delay discounting and associated changes in brain activity.

**Method:**

A total of 102 female participants (AUD, 27; bulimia nervosa, 25; healthy controls, 50) underwent repeated functional magnetic resonance imaging scanning. Initially, all participants performed a monetary delay discounting task (DDT), followed by a food or alcohol DDT before and after stress induction. Specifically, those with bulimia nervosa completed a food DDT, those with AUD completed an alcohol DDT and healthy controls were randomly allocated to one or either DDT.

**Results:**

Participants with AUD, but not healthy controls, displayed a higher discounting of alcohol after stress. Healthy controls, but not those with bulimia nervosa, had nominally higher discounting rates of food following stress, although not significant following multiple testing correction. Participants with AUD displayed a lower activity of the right supplementary motor area while discounting alcohol after stress. Healthy controls showed a lower activity of the frontal cortex and a higher activity of the motor cortex while discounting food after stress, while those with bulimia nervosa displayed a higher activity of the occipital cortex.

**Conclusions:**

The results suggest that, in subjects with AUD, stress induces neurobiological changes that cause them to prefer more immediately available alcohol. However, the results observed in participants with bulimia nervosa suggest a more complex relation between stress and food.

Both bulimia nervosa and alcohol use disorder (AUD) are characterised by binge behaviour (i.e. binge eating and binge drinking), where large amounts of a substance (i.e. food and alcohol, respectively) are consumed within a short period of time.^1^ Although treatments for bulimia nervosa and AUD exist, large numbers of individuals are not able to abstain from binge eating or binge drinking following treatment.^2,3^ More effective interventions are therefore needed but, in order to develop these, a better understanding of what triggers binge behaviour is required. Most studies have investigated bulimia nervosa and AUD separately to explore these triggers; however, because these conditions share a number of similarities, studying them together could provide more information by identifying both common and unique factors. One factor that is thought to play a role in both bulimia nervosa and AUD is delay discounting, which is the process whereby rewards decrease in value the more delayed they are.^4^ This means that individuals usually prefer more immediately available rewards over delayed ones.^4^

From a behavioural standpoint, delay discounting involves both a reward-processing and an impulsive-like component.^5,6^ On the one hand, delay discounting is subsumed under the positive valence systems of the Research Domain Criteria (RDoC), where it is regarded as a moderator of reward valuation.^5^ On the other hand, delay discounting is described as a distinct construct of impulsive-like behaviour because it reduces the significance of negative consequences in the distant future, making it more likely for individuals to engage in behaviours that provide immediate gratification.^6^ With regard to bulimia nervosa and AUD, it is thought that individuals display a steeper delay discounting of rewards, leading them to put more value on short-term benefits than long-term negative consequences. Delay discounting can be investigated using a delay discounting task (DDT), where participants need to choose between a smaller earlier reward and a larger later reward.^4^ Based on the decisions made by an individual, a delay discounting rate can be calculated where higher values represent a stronger preference for more immediate rewards.^4^ Previous studies show that those with bulimia nervosa and AUD display higher delay discounting rates for monetary rewards, meaning that they prefer a more immediately available amount of money than a larger delayed amount.^7,8^ However, with regard to disorder-specific food and alcohol delay discounting, only a few studies have been published.^9,10^ We identified one study that investigated alcohol delay discounting in AUD, which found higher discounting rates compared with healthy controls.^9^ However, studies in individuals without AUD report that preferring larger amounts of alcohol during a DDT predicts past-month adverse consequences of drinking, and that individuals who meet more DSM-5 criteria for AUD, or score higher on the Alcohol Use Disorders Identification Test (AUDIT), also prefer more immediately available alcohol.^11–13^ We identified one study that investigated food delay discounting in bulimia nervosa, but that study found lower discounting rates.^10^ In summary, those with bulimia nervosa or AUD seem to display a higher delay discounting of money, subjects with AUD may have higher delay discounting rates for alcohol and those with bulimia nervosa could actually have lower delay discounting rates for food, although further research on food and alcohol delay discounting in individuals with bulimia nervosa or AUD is needed.

From a neurobiological standpoint, one model suggests that delay discounting is processed in five subsequent steps involving specific brain regions at each step;^14^ an overview can be seen in Fig. 1. The key steps are III and IV, corresponding to the attribution of subjective value to the earlier and delayed rewards and the comparison between them. The attribution of subjective value is thought to be performed by the anterior cingulate cortex (ACC), posterior cingulate cortex (PCC), middle frontal gyrus (MFG), orbitofrontal cortex (OFC), insula, nucleus accumbens (NAc) and caudate nucleus.^14^ The comparison between these subjective values is thought to be performed by a dual system, consisting of a beta (β)-system that is impulsive, reflexive and focused on the immediate reward, and a delta (δ)-system that is controlled and processes both immediate and delayed rewards.^14,15^ The β-system is thought to be represented in the ACC and OFC, while the δ-system is encoded in the dorsomedial prefrontal cortex (dmPFC) and dorsolateral prefrontal cortex (dlPFC).^14,15^ In regard to the functioning of these brain regions during a DDT, no study to date has compared current subjects with bulimia nervosa and healthy controls. However, a study in remitted patients with bulimia nervosa found lower activity of the caudate nucleus during a monetary DDT after fasting, but higher activity after eating.^16^ More studies have been performed in those with AUD. Here, studies report that individuals display a greater deactivation of the superior frontal gyrus (SFG) and PCC when making impulsive monetary choices, but a greater activation of the dlPFC, (pre)cuneus, insula and OFC when choosing the delayed option.^17–19^ Furthermore, one study in heavy drinkers found that left mOFC activity during an alcohol/money DDT correlated with alcohol-induced craving for alcohol.^20^ In summary, studies indicate that delay discounting for money could be processed differently in subjects with bulimia nervosa and AUD, although it has not been sufficiently explored whether this is also the case for food or alcohol.

**Fig. 1:**
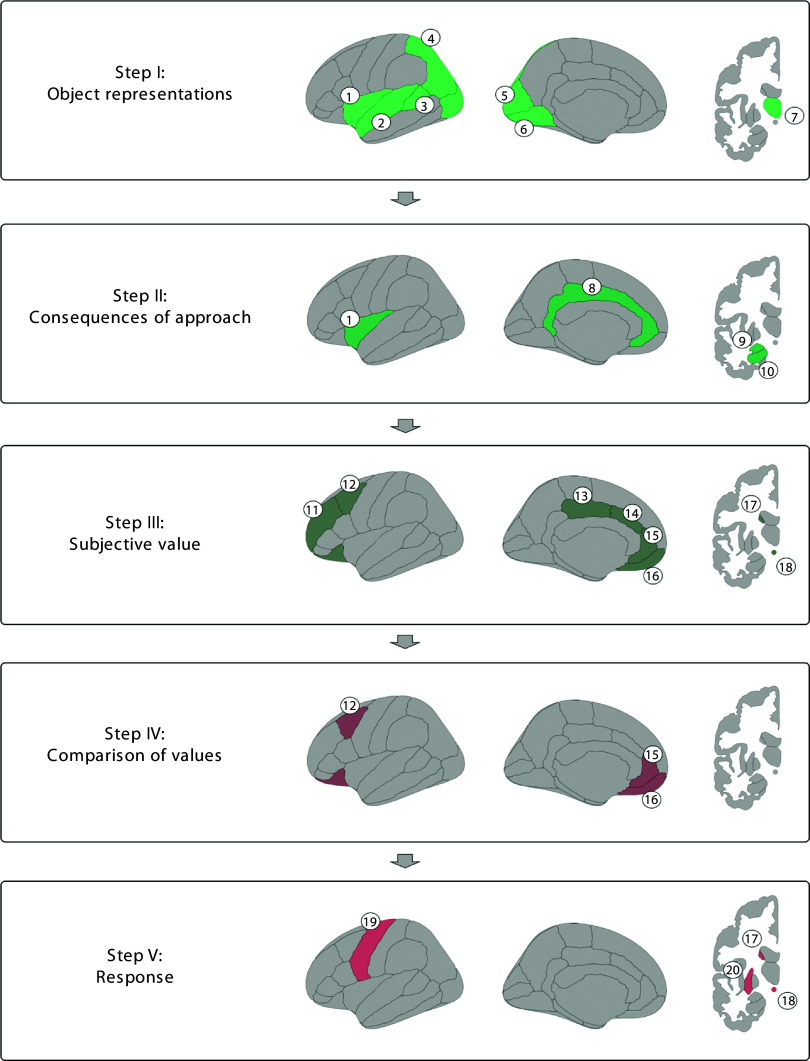
Neural processing of delay discounting. First, sensory information is transformed into object representations. Second, the object representations are used to establish the consequences of choosing the earlier or delayed reward. Third, the consequences are attributed a subjective value. Fourth, the subjective value between the earlier and delayed reward is compared using a dual system. Fifth, information on the decision is used to produce motor responses to acquire the reward. Regions: 1, insula; 2, superior temporal gyrus; 3, angular gyrus; 4, parietal cortex; 5, occipital cortex; 6, lingual gyrus; 7, thalamus; 8, cingulate cortex; 9, amygdala; 10, hippocampus; 11, middle frontal gyrus; 12, dorsolateral prefrontal cortex; 13, posterior cingulate gyrus; 14, anterior cingulate gyrus; 15, ventromedial prefrontal cortex; 16, orbitofrontal cortex; 17, caudate nucleus; 18, nucleus accumbens; 19, precentral gyrus; 20, putamen.

Furthermore, although these findings indicate that there are differences in delay discounting between participants and controls, they raise the question as to how these differences lead to periods of binge eating or binge drinking in subjects with bulimia nervosa or AUD. A key factor in this process could be stress, which is thought to be important in both bulimia nervosa and AUD, with most theoretical models positing that binge eating and binge drinking can be a way for the individual to cope with stress.^21,22^ It could be hypothesised that stress induces functional brain changes in people, resulting in an increase in delay discounting, making them see the short-term benefits of coping through eating or drinking alcohol as more valuable than the long-term benefits of remission. Indeed, studies in healthy volunteers found that acute stress increases the delay discounting of money and makes individuals choose based more on subjective value, although one study in heavy drinkers did not replicate the effect of stress on delay discounting of money.^23–26^ However, although these studies indicate that stress could increase the delay discounting of money, its impact on food or alcohol delay discounting is unclear.

In summary, individuals with bulimia nervosa or AUD may display differences in the delay discounting of money, food or alcohol, as well as in how delay discounting is processed in the brain, which could be further amplified under stress. However, there are important gaps in the literature, which is why this study investigated the following hypotheses: (a) subjects with bulimia nervosa and AUD display higher delay discounting rates than healthy controls for money; (b) individuals with bulimia nervosa or AUD, but not healthy controls, display higher delay discounting rates for food and alcohol; and (c) stress increases delay discounting rates for food and alcohol in people with bulimia nervosa and AUD, respectively, but not in healthy controls. Although one previous study found lower delay discounting rates for food in individuals with bulimia nervosa, the current study still hypothesises that those with bulimia nervosa display higher delay discounting rates for food, based on studies looking at delay discounting of money in subjects with bulimia nervosa, and on the theory that these individuals prefer more short-term food rewards.^4,10,27^ Additionally, differences in delay discounting between healthy controls and those with bulimia nervosa or AUD are hypothesised to be associated with brain activity changes in regions involved in the attribution and comparison of subjective value (i.e. the ACC, PCC, MFG, OFC, insula, dmPFC, dlPFC, NAc and CN).

## Method

### Participants

A total of 102 female right-handed participants were included in the study (AUD, 27; bulimia nervosa, 25; healthy controls, 50) following removal of 4 participants (bulimia nervosa, 3; healthy controls, 1) due to artefacts and incidental findings. Recruitment ran from September 2019 to February 2022 (eMethods 1 available in the Supplementary material at https://doi.org/10.1192/bjo.2025.10821). The full inclusion and exclusion criteria can be found in the Supplementary materials (eMethods 2). Importantly, patients needed to meet the criteria for bulimia nervosa or AUD of DSM-5, and having a maximum illness duration of 5 years.^1^ This maximum illness duration was set for the reason that the role of impulsive-like behaviour is thought to be greatest in the first years following the onset of bulimia nervosa or AUD, because binge eating and binge drinking become more habitual over time.^21,27^ Participants with AUD also needed to display a pattern of repetitive binge drinking according to the criteria of the National Institute on Alcohol Abuse and Alcoholism (i.e.,drinking 4 units of alcohol within 2 h for women).^28^ Patients could not meet the criteria for both AUD and bulimia nervosa, because the inclusion of a group with both would not be compatible with the design of the DDTs as described below (i.e. performing either a food or alcohol DDT). All participants gave their written consent, and the study was approved by the local ethical committee of UZ/KU Leuven.

### Procedure

**Fig. 2:**
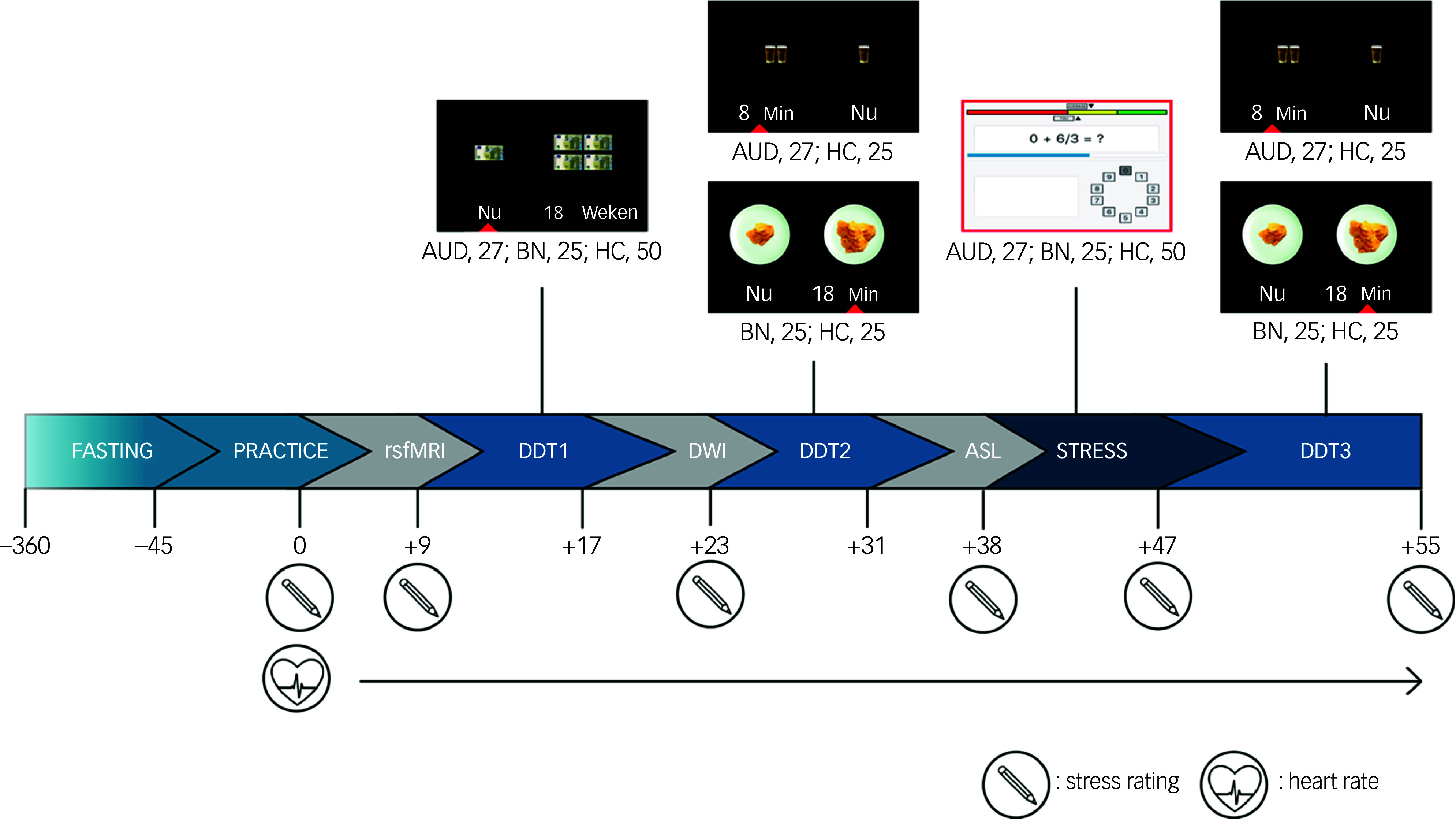
Study design. Participants fasted in the 6 h prior to the magnetic resonance imaging scan. They came in 45 min early to practise the tasks. The scan was divided into four main parts. First, all participants performed a monetary delay discounting task (DDT1). Second, those with bulimia nervosa completed a DDT with food while those with alcohol use disorder (AUD) completed one with alcohol. Healthy controls (HC) were randomly allocated to either the food or alcohol DDT (DDT2 pre-stress). Third, stress was induced with the Montreal Imaging Stress Task (MIST; STRESS). Fourth, participants repeated the food or alcohol DDT (DDT3 post-stress). During the scan, participants reported on their stress level; their heart rate was measured with a photoplethysmography sensor. ASL, arterial spin labelling; BN, bulimia nervosa; DWI, diffusion-weighted imaging; Nu, now; rsfMRI, resting-state functional magnetic resonance imaging; Weken, weeks.

The procedure of the magnetic resonance imaging (MRI) scanning can be seen in Fig. 2. Participants were instructed not to eat or drink anything in the 6 h leading up to the scan, and needed to refrain from using substances (e.g. alcohol, cannabis, etc.) in the 24 h before the scan. The median interquartile range (IQR) starting time of the scan was 17:00 (16:00–18:00). Participants were asked whether they had adhered to these instructions on the day of the scanning procedure and, if not, the scan was rescheduled. This was the case for one individual. Participants came in 45 min early to familiarise themselves with the tasks of the study in a practice session. Immediately before scanning, a photoplethysmography (PPG) sensor was placed on the left index finger to measure heart rate. The scan itself was divided into four main parts. First, all participants performed a monetary DDT (DDT1), followed by a disorder-specific (e.g. food or alcohol) DDT (DDT2). This meant that participants with bulimia nervosa completed a DDT with food while those with AUD completed one with alcohol. Healthy controls were randomly allocated to either the food (healthy controls_food_) or alcohol (healthy controls_alcohol_) DDT as a comparison for those with bulimia nervosa and AUD, respectively. Third, stress was induced using the Montreal Imaging Stress Task (MIST).^29^ Fourth, participants repeated the food or alcohol DDT post-MIST (DDT3). The DDT1, DDT2 and STRESS blocks were separated by other MRI sequences not analysed in this manuscript. Further information on the study procedure can be found in the supplementary materials (eMethods 3 and eFig. 1).

### Measures

#### Baseline measures

The Structured Clinical Interview for DSM-5 (SCID-5-S) was used to confirm the diagnosis of bulimia nervosa or AUD and to screen for other psychiatric disorders.^30^ Bulimia nervosa and AUD severity were assessed with the Eating Disorder Examination Questionnaire (EDE-Q) and AUDIT, respectively.^31,32^ EDE-Q had excellent internal consistency, with a Cronbach’s alpha of 0.95, and AUDIT had good internal consistency, with Cronbach’s alpha of 0.89. Of those participants with bulimia nervosa, 19 (76%) had an EDE-Q score over the clinical cut-off (2.8); among those with AUD, 26 (96%) had an AUDIT score over the threshold of medium-level problems (8) and 13 (48%) had an AUDIT score over the threshold of high-level problems (15). Additionally, using the DSM-5 criteria, there were 7 (26.9%) participants with mild AUD (2–3 positive criteria), 9 (34.6%) with moderate AUD (4–5 positive criteria) and 8 (30.8%) with severe AUD (>6 positive criteria). The Depression Anxiety Stress Scale (DASS) was used to inventorise depression, anxiety and stress symptoms.^33^ This clinical assessment took place on a separate study visit before functional MRI (fMRI) scanning. The median (IQR) number of days between clinical assessment and fMRI scanning was 15 (9–42) days.

#### Delay discounting tasks

The DDTs were adapted from a food DDT that was used in a previous study.^34^ In each DDT, the participants chose between an amount of money, food or alcohol that was immediately available and a larger amount of the same reward that was available following a delay. The immediate rewards were either 5 euros, around 250 kcal of food or 1 unit of alcohol, while the delayed rewards were multiples (2- to 5-fold) of the immediate reward. The rewards were hypothetical, which is a valid alternative for real rewards in studies on delay discounting.^35^ The type of food and alcohol used in the DDT (one type each for the DDT before and after stress) was selected by each participant from a list of possible food items and alcoholic beverages in the practice session (eMethods 4). Each of the multiples was paired with 1 of 10 delays for each decision, resulting in 40 trials per DDT. These delays were the deciles of a maximally tolerated delay level plus 10%, which was determined in the practice session (eMethods 5). Delays for the DDT with money were expressed in weeks, while those for the DDTs with food and alcohol were expressed in minutes. This is similar to previous studies, and was done so that the choices concerning food and alcohol more closely resembled those of people in evertday life.^36^ Each trial started with an inter-stimulus interval (ISI) that varied between 3.5 and 5.0 s. Next, participants were shown the immediate and delayed options and were allowed 6 s to make their choice using a button box. A red arrowhead then appeared beneath their chosen option for the remainder of the 6 s before the next trial started. The ISI, as well as the magnitude, delay and position of the delayed reward, were determined pseudo-randomly for each trial. The total duration of each DDT (money, food or alcohol before stress, and food or alcohol after stress) was 6 min and 50 s.

#### MIST

MIST is a task that uses mental arithmetic, failure and negative social evaluation to induce stress in participants.^29,37^ It typically consists of a rest condition (i.e. only the interface), a control condition (i.e. only mental arithmetic) and an experimental condition (i.e. mental arithmetic with the stress components). Because the purpose of using MIST in this study was to induce stress, participants completed only the experimental condition with the scanner. Under this condition, participants were given mathematical problems and were required to respond before a certain amount of time had expired. Participants saw their own performance and a fictive average performance of all previously included subjects. They were then instructed to better this average, but the task adapted the difficulty of the mathematical problems so that participants performed poorly. In addition, negative feedback was given to participants emphasising their poor performance and urging them to perform better. The difficulty level for each participant with the scanner was established in the practice session (eMethods 6).^38^ The total duration of MIST in the scanner was 6 min.

#### Subjective stress

Participants rated their stress levels at the beginning of the scan, before each task (DDT1, DDT2, STRESS, DDT3) and at the end of the scan with a visual analogue scale (VAS). This VAS had 10 levels, ranging from 0 (not stressed at all) to 10 (never experienced such stress before).

#### Heart rate

PPG data were gathered at 500 Hz with the wireless pulse oximeter of the MRI system. These were then preprocessed with SCANPHYSLOG_Tools (Matlab R2019a).^39^ First, peaks were identified in the pulse waveforms. Second, the data were divided into 1-min epochs and heart rate for each epoch was calculated. Third, implausible heart rates below 30/min or above 200/min were filtered out.

### MRI sequences

Scanning was performed on a 3T Achieva dStream Philips MRI scanner with a 32-channel receiver head coil. T2*-weighted echo-planar images were acquired during every DDT (275 volumes, 46 slices, repetition time 1.5 s, echo time 33 ms, flip angle 80°, voxel size 2.14 × 2.14 × 3.00 mm^3^, multiband acceleration factor 2, acquisition plane axial). A high-resolution, T1-weighted image was acquired during MIST using a three-dimensional turbo field echo sequence (208 slices, repetition time 5.9 ms, echo time 2.7 ms, flip angle 8°, voxel size 0.8 × 0.8 × 0.8 mm^3^, acquisition plane axial).

### Data analysis

The data were analysed and reported in accordance with the guidelines of Frank et al,^40^ which were specifically developed to help set up and analyse neuroimaging studies that include patients with an eating disorder. A checklist can be found in the Supplementary material. The data are available upon request.

#### Baseline group comparisons

For continuous baseline measures, differences between groups were evaluated using either a *t*-test or Wilcoxon rank sum test, depending on whether the variable followed a normal distribution; normality was evaluated using the Kolmogorov–Smirnov test. For categorical baseline measures, Fisher’s exact test was used. These analyses were conducted separately for participants with AUD or bulimia nervosa and their respective control groups.

#### Delay discounting

For every DDT, a *k*-value (i.e. a delay discounting rate) was estimated by fitting the choice data to a hyperbolic discounting model (Matlab R2019a for iOS; MathWorks, Natick, MA, USA; https://www.mathworks.com/products/matlab.html) (eMethods 7).^34^ The *k*-value describes how steeply value is degraded by delay, and is widely used in the literature to study delay discounting.^4,41^ The *k*-values were logarithmically transformed due to their non-normal distribution. The log(*k*)-values at each DDT were compared between groups with robust linear regression models. These models included the log(*k*)-values as the outcome and included group as the main effect (bulimia nervosa, AUD, healthy controls for the monetary DDT; bulimia nervosa, healthy controls_food_ for the food DDT before and after stress; AUD, healthy controls_alcohol_ for the alcohol DDT before and after stress). The impact of stress was evaluated within groups with robust linear mixed models. These models included random intercepts for the participants, the log(*k*)-values of the disorder-specific DDT as the outcome as well as group (bulimia nervosa, healthy controls_food_ for the food DDT; AUD, healthy controls_alcohol_ for the alcohol DDT) and time (before, after MIST) as main and interaction effects. All models included age and body mass index (BMI) as covariates. The model results were corrected for multiple comparisons using the Benjamini–Hochberg procedure. Corrections were applied separately for AUD versus control comparison (six tests) and bulimia nervosa versus control comparison (six tests). Findings with *P*_corrected_ < 0.05 were considered significant, while those with *P*_corrected_ > 0.05 and *P*_uncorrected_ < 0.05 were considered nominally significant.

#### Subjective and physiological stress response

The impact of MIST on subjective stress ratings and heart rate was evaluated with robust linear mixed models, similar to the models described above, but these included either subjective stress ratings or heart rate as outcome. For subjective stress, only the data pre- and post-MIST were used. For heart rate, only the data from the 6 min pre-MIST (i.e. during a resting-state arterial spin labelling sequence) and 6 min during the MIST were used. The results from the models were corrected for multiple testing using the Benjamini–Hochberg procedure. This was done separately for the results concerning heart rate (five tests) and subjective stress (five tests). Findings with *P*_corrected_ < 0.05 were considered significant, while those with *P*_corrected_ > 0.05 and *P*_uncorrected_ < 0.05 were considered nominally significant.

#### fMRI data

The fMRI data of each DDT were initially preprocessed with fmriprep (version 21.0.1, iOS; https://fmriprep.org/en/stable/), after which they were smoothed with an 8-mm, full-width at half- maximum (FWHM) Gaussian kernel in SPM12 (Matlab R2019a for iOS; Functional Imaging Laboratory, London, UK; https://www.fil.ion.ucl.ac.uk/spm/software/spm12/) (eMethods 8).^42^ These smoothed images were then used in a first-level analysis in SPM12 (eMethods 9). On the one hand, this analysis included two boxcar regressors that separately modelled the decision and feedback stages. The decision stages started with the presentation of the rewards and ended when participants submitted their choice through the response box. The feedback stages followed immediately afterwards and ended 6 s following the initial presentation of the rewards. These boxcar regressors were convolved with the canonical haemodynamic response function. On the other hand, the first-level analysis included three rotation, three translation, six derivatives, five wCompCor, five cCompCor and five cosine variables as nuisance regressors.^43–45^ Further information on the nuisance regressors can be found in the Supplementary material (eMethods 8). From the first-level analysis, contrast images were calculated for the decision stages (i.e. decision stage, 1; feedback stage, 0).

These contrast images were used in a second-level analysis in SPM12 (Matlab R2019a). First, whole-brain analyses compared brain activity at each DDT between groups. A whole-brain analysis was chosen rather than a region-of-interest-based analysis, because this study is the first to investigate the effect of stress on brain activity during delay discounting, and due to the large number of brain areas involved in delay discounting. This was carried out with an analysis of variance design (group: bulimia nervosa, AUD, healthy controls) for the monetary DDT and a *t*-test design for the food (group: bulimia nervosa, healthy controls_food_) or alcohol (group: AUD, healthy controls_alcohol_) DDTs before and after stress. Second, whole-brain analyses investigated the impact of stress on brain activity during the food or alcohol DDT within groups. This was performed with a full factorial design that included both group (bulimia nervosa, healthy controls_food_ for the food DDT; AUD, healthy controls_alcohol_ for the alcohol DDT) and time (before, after MIST) as main and interaction effects. All designs included age and BMI as covariates of no interest, because these factors have a significant impact on neuroimaging, with age also being recommended as a covariate in fMRI analyses by Frank et al,^40^ and due to the lack of an active matching procedure for age and BMI.^46,47^ The two-tailed statistical contrasts were tested for significance using cluster-level inference with an uncorrected cluster-defining threshold of *P* < 0.001 and a family-wise error (FWE)-corrected cluster threshold of *P* < 0.05. Third, underlying contrast values of significant clusters were extracted with the MarsBaR toolbox (Matlab R2019a) and related to relevant participant characteristics. As advised by the guidelines given in Frank et al, the contrast values were related to the log(*k*)-values, AUDIT and EDE-Q scores, binge eating and binge drinking frequency, age, BMI and use of contraceptives.^40^ An exploration of the effect of ethnicity, menstrual cycle, presence of comorbidities or history of anorexia nervosa was not possible due to a lack of observations. An exploration of illness duration was also not performed due to the restricted range in the current study. The analyses were performed with robust regression models using the robustlmm package (R, version 4.4.1 for iOS; R Foundation, Vienna, Austria; https://www.r-project.org/), which included the contrast values as the outcome and included a patient characteristic as predictor. Because the whole-brain analysis included age and BMI as covariates, these variables were also entered as covariates in the robust regression models. For this reason, the relations between the contrast values and age or BMI were investigated with one model that included both age and BMI as predictors.

## Results

### Sample characteristics

The characteristics of participants with bulimia nervosa (*n* = 25) and AUD (*n* = 27) and their respective controls (healthy controls_food_, *n* = 25 and healthy controls_alcohol_, *n* = 25) are provided in Table 1. The characteristics of the pooled healthy controls group (*n* = 50) are provided in the Supplementary materials (eTable 1). There were no significant differences in age, BMI, years of education or ethnicity between participants and their respective control groups. Notably, patients with bulimia nervosa had higher scores on the depression (mean [s.d.] bulimia nervosa 10.6 [8.8], mean [s.d.] healthy controls_food_ 2.6 [2.4], *P* < 0.001), anxiety (mean [s.d.] bulimia nervosa 6.6 [6.9], mean [s.d.] healthy controls_food_ 2.1 [2.1], *P* = 0.008) and stress (mean [s.d.] bulimia nervosa 12.9 [9.0], mean [s.d.] healthy controls_food_ 6.0 [4.1], *P* = 0.005) subscales of the DASS-42, as well as higher total scores (mean [s.d.] bulimia nervosa 30.1 [21.1], mean [s.d.] healthy controls_food_ 10.7 [6.8], *P* < 0.001).

**Table 1:** Sample characteristics

Parameters	AUD (*n* = 27)	Healthy controls_alcohol_ (*n* = 25)	*P*-value	Healthy controls_food_ (*n* = 25)	BN (*n* = 25)	*P*-value
	Mean (s.d.)			Mean (s.d.)		
Age (years)	21.7 (4.6)	21.0 (1.9)	0.970	22.2 (3.0)	23.0 (4.5)	0.822
BMI	22.4 (2.1)	22.1 (1.6)	0.564	22.5 (2.7)	25.5 (5.8)	0.084
Illness duration (years)	3.0 (1.2)	0 (0)	<0.001^b^	0 (0)	2.4 (1.5)	<0.001^b^
Education (years)	14.6 (1.8)	14.7 (1.2)	0.904	15.6 (1.9)	15.0 (2.0)	0.316
AUDIT	13.9 (4.4)	3.6 (2.1)	<0.001^b^	3.5 (2.1)	4.1 (3.6)	0.953
EDE-Q						
Restraint	0.8 (1.0)	0.3 (0.6)	0.070	0.5 (0.8)	3.0 (1.5)	<0.001^b^
Shape concern	1.7 (1.5)	0.9 (0.8)	0.029^b^	1.1 (1.1)	4.3 (1.4)	<0.001^b^
Weight concern	1.3 (1.4)	0.8 (0.9)	0.146	0.7 (1.0)	4.1 (1.3)	<0.001^b^
Eating concern	0.5 (0.9)	0.2 (0.2)	0.204	0.3 (0.5)	2.9 (1.6)	<0.001^b^
Total	1.2 (1.1)	0.6 (0.5)	0.057	0.7 (0.8)	3.7 (1.2)	<0.001^b^
Eating disorder symptoms (days/4 weeks)						
Binge eating	0 (0)	0 (0)	>0.999	0 (0)	10.1 (8.5)	<0.001^b^
Fasting	0 (0)	0 (0)	>0.999	0 (0)	6.6 (7.8)	<0.001^b^
Vomiting	0 (0)	0 (0)	>0.999	0 (0)	4.3 (8.9)	<0.001^b^
Laxative use	0 (0)	0 (0)	>0.999	0 (0)	0.6 (5.6)	<0.001^b^
Diuretic use	0 (0)	0 (0)	>0.999	0 (0)	1.1 (5.6)	<0.001^b^
Compensatory exercise	0 (0)	0 (0)	>0.999	0 (0)	6.1 (6.4)	<0.001^b^
DASS-42						
Total	22.8 (20.8)	13.5 (12.5)	0.113	10.7 (6.8)	30.1 (21.1)	<0.001^b^
Anxiety	5.7 (6.8)	3.1 (4.1)	0.112	2.1 (2.2)	6.6 (6.9)	0.008^b^
Depression	6.1 (6.9)	3.3 (3.9)	0.180	2.6 (2.4)	10.6 (8.8)	<0.001^b^
Stress	10.9 (9.2)	7.1 (5.6)	0.155	6.0 (4.1)	12.9 (9.0)	0.005^b^
	*n* (%)	*P*-value	*n* (%)	*P*-value	*n* (%)	*P*-value
Binge drinking frequency						
Never	0 (0)	12 (48)	<0.001^b^	14 (56)	13 (52)	0.836
Annually	0 (0)	1 (4)		2 (8)	4 (16)	
Semi-annually	0 (0)	3 (12)		3 (12)	1 (4)	
Three-monthly	3 (11)	5 (20)		4 (16)	4 (16)	
Monthly	6 (22)	3 (12)		2 (8)	2 (8)	
Biweekly	12 (44)	1 (5)		0 (0)	0 (0)	
Weekly	3 (11)	0 (0)		0 (0)	1 (4)	
>Weekly	3 (11)	0 (0)		0 (0)	0 (0)	
Therapy (BN/AUD)						
Past	0 (0)	0 (0)	>0.999	0 (0)	10 (40)	0.001^b^
Present^a^	0 (0)	0 (0)	>0.999	0 (0)	4 (16)	0.110
Previous AN	0 (0)	0 (0)	>0.999	0 (0)	6 (24)	0.022^b^
Ethnicity						
Caucasian	26 (96)	25 (100)	>0.999	23 (92)	24 (96)	>0.999
Latino	1 (4)	0 (0)		0 (0)	0 (0)	
Asian	0 (0)	0 (0)		1 (4)	0 (0)	
Mixed	0 (0)	0 (0)		1 (4)	0 (0)	
Middle-Eastern	0 (0)	0 (0)		0 (0)	1 (4)	
Contraceptive use	21(78)	22 (88)	0.469	24 (96)	19 (76)	0.098
Amenorrhea	0 (0)	0 (0)	>0.999	0 (0)	1 (4)	>0.999
SSRI	3 (11)	0 (0)	0.236	0 (0)	4 (16)	0.110
Comorbidities						
MDD	1 (4)	0 (0)	0.723	0 (0)	1 (4)	0.235
PD	1 (4)	0 (0)		0 (0)	1 (4)	
SAD	1 (4)	0 (0)		0 (0)	1 (4)	
PTSD	1 (4)	0 (0)		0 (0)	0 (0)	

AN, anorexia nervosa; AUD, alcohol use disorder; AUDIT, alcohol use disorders identification test; BMI, body mass index; BN, bulimia nervosa; DASS-42, Depression Anxiety Stress Scale; EDE-Q, Eating Disorder Examination Questionnaire; MDD, major depressive disorder; PD, panic disorder; PTSD, post-traumatic stress disorder; SAD, social anxiety disorder; SSRI, selective serotonin reuptake inhibitors.

a Patients were in different treatment modalities (i.e. ambulatory psychologist, psychiatrist, dietician or out-patient treatment programme).

b Significant result.

### Behavioural data

Results for the statistical tests for DDTs can be found in Table 2. A summary of the log(*k*)-values from the DDTs can be found in the Supplementary material (eTable 2).

**Table 2:** Differences in delay discounting

Model	Effect	*β*	s.e.	*P*_uncorrected_	*P*_corrected_
Delay discounting money	Group (AUD versus healthy controls)	0.134	0.122	0.271	0.325
	Group (BN versus healthy controls)	0.137	0.135	0.311	0.574
Delay discounting alcohol before MIST	Group (AUD versus HC_alcohol_)	−0.357	0.191	0.068	0.136
Delay discounting alcohol after MIST	Group (AUD versus HC_alcohol_)	−0.253	0.184	0.174	0.260
Delay discounting alcohol after versus before MIST	Group (AUD)	0.073	0.019	<0.001^b^	0.002^b^
	Group (HC_alcohol_)	0.006	0.019	0.761	0.761
Delay discounting food before MIST	Group (BN versus HC_food_)	−0.061	0.121	0.613	0.613
Delay discounting food after MIST	Group (BN versus HC_food_)	−0.098	0.132	0.416	0.574
Delay discounting food after versus before MIST	Group (BN)	0.020	0.028	0.478	0.574
	Group (HC_food_)	0.060	0.028	0.039^a^	0.233

AUD, alcohol use disorder; *β*, estimate; BN, bulimia nervosa; HC, healthy control; HC_alcohol_, healthy controls who performed the alcohol delay discounting task; HC_food_, healthy controls who performed the food delay discounting task; MIST, Montreal Imaging Stress Task; *P*_uncorrected_, uncorrected *P*-value; *P*_corrected_, corrected *P*-value using the Benjamini–Hochberg procedure.

a Nominally significant result.

b Significant result.

#### Delay discounting of money

There were no significant differences between the log(*k*)-values of the different groups (AUD versus bulimia nervosa; AUD versus healthy controls; bulimia nervosa versus healthy controls).

#### Delay discounting of food

There were no significant differences between the log(*k*)-values of participants with bulimia nervosa and healthy controls_food_ before MIST. There were nominally higher log(*k*)-values following MIST in healthy controls_food_ compared with pre-MIST (*β* = 0.060, s.e. = 0.028, *P*_uncorrected_ = 0.039, *P*_corrected_ = 0.233), but this difference did not remain significant after correcting for multiple testing. There was no significant difference in log(*k*)-values following MIST in participants with bulimia nervosa (*β* = 0.020, s.e. = 0.028, *P*_corrected_ = 0.574) compared with pre-MIST. Furthermore, there was no significant interaction effect between MIST task and group concerning log(*k*)-values. There were no significant differences between the log(*k*)-values of participants with bulimia nervosa and healthy controls_food_ following MIST.

#### Delay discounting of alcohol

There were no significant differences between the log(*k*)-values of participants with AUD and healthy controls_alcohol_ before MIST. Compared with pre-MIST, there were significantly higher log(*k*)-values post-MIST in participants with AUD (*β* = 0.073, s.e. = 0.19, *P*_corrected_ = 0.002), but not in healthy controls_alcohol_ (*β* = 0.006, s.e. = 0.019, *P*_corrected_ = 0.761). In other words, participants with AUD chose the immediately available alcohol more often following the induction of stress. There was a significant interaction effect between MIST task and group concerning log(*k*)-values, with participants with AUD having a significantly higher increase in values compared with healthy controls_alcohol_ (*β* = 0.067, s.e. = 0.027, *P*_corrected_ = 0.049).

#### Subjective and physiological stress response

There was a significant increase in subjective stress ratings for all groups post-MIST compared with pre-MIST (healthy controls *β* = 3.369, s.e. = 0.270, *P*_corrected_ < 0.001; bulimia nervosa *β* = 4.654, s.e. = 0.381, *P*_corrected_ < 0.001; AUD *β* = 4.335, s.e. = 0.367, *P*_corrected_ < 0.001), but this was more pronounced in patients (bulimia nervosa *β* = 1.30, s.e. = 0.467, *P*_corrected_ = 0.008; AUD *β* = 0.967, s.e. = 0.456, *P*_corrected_ = 0.036). There was also a significant increase in heart rate during MIST compared with pre-MIST in all groups (healthy controls *β* = 10.084, s.e. = 0.613, *P*_corrected_ < 0.001; bulimia nervosa *β* = 10.416, s.e. = 0.857, *P*_corrected_ < 0.001; AUD *β* = 8.077, s.e. = 0.872, *P*_corrected_ < 0.001), but this did not differ significantly between groups.

### fMRI data

**Fig. 3:**
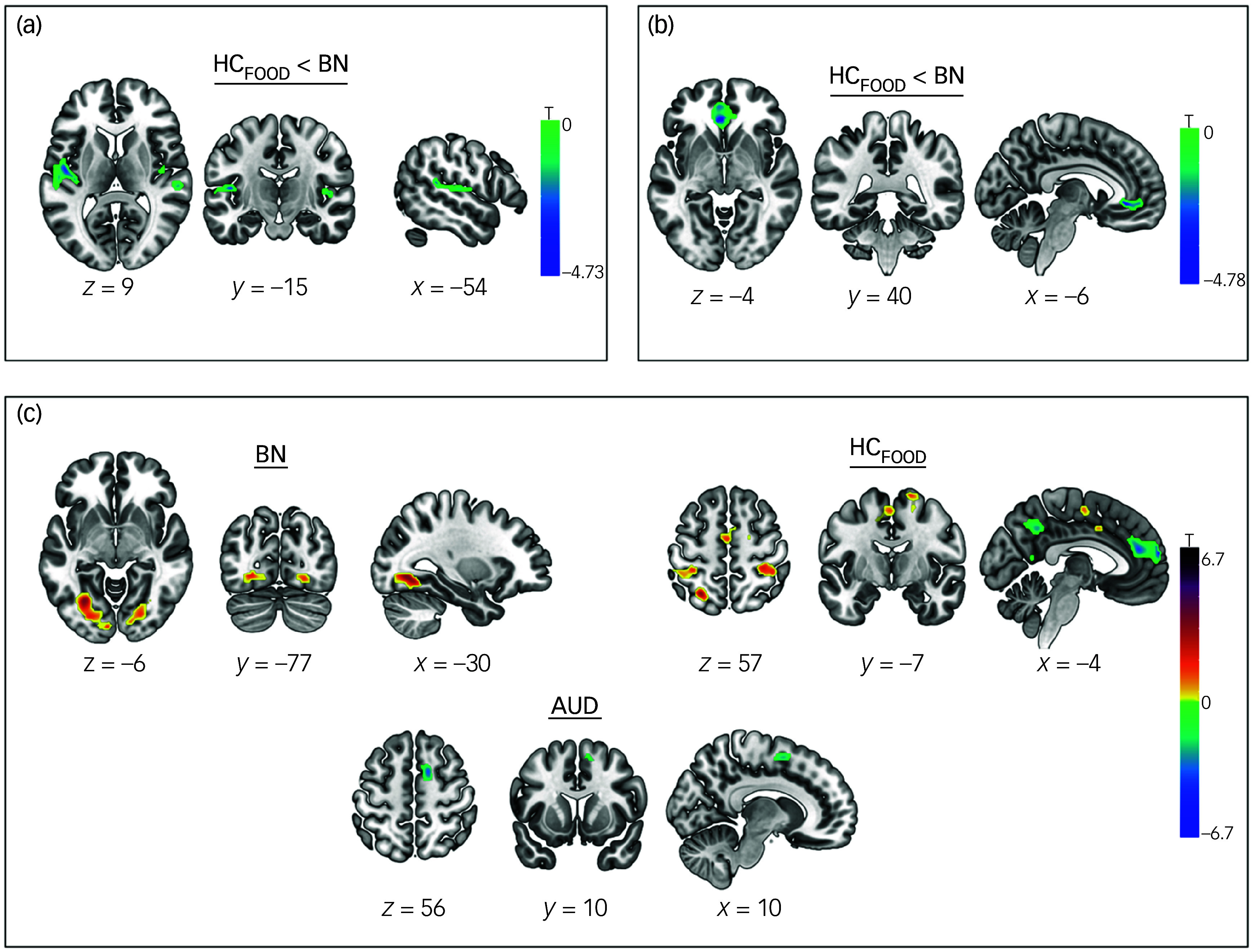
Whole-brain between- and within-group differences during the delay discounting tasks (DDTs). (a) During the food DDT before the Montreal Imaging Stress Task (MIST, pre-stress), participants with bulimia nervosa (BN) showed a weaker deactivation of the left insula and right insula compared with healthy controls_FOOD_ (HC_FOOD_); (b) during the food DDT following MIST (post-stress), those with BN displayed a weaker deactivation of the anterior cingulate cortex compared with HC_FOOD_; (c) post-MIST, compared with pre-MIST, participants with BN displayed a higher activity of the left occipital cortex and right occipital cortex. HC_FOOD_ showed higher activity of the left and right postcentral gyrus and left and right supplementary motor area, but lower activity of the middle and superior frontal gyrus and posterior cingulate cortex. Participants with alcohol use disorder (AUD) displayed lower activity of the right supplementary motor area. HC_FOOD_, healthy controls who performed the food DDT.

**Fig. 4:**
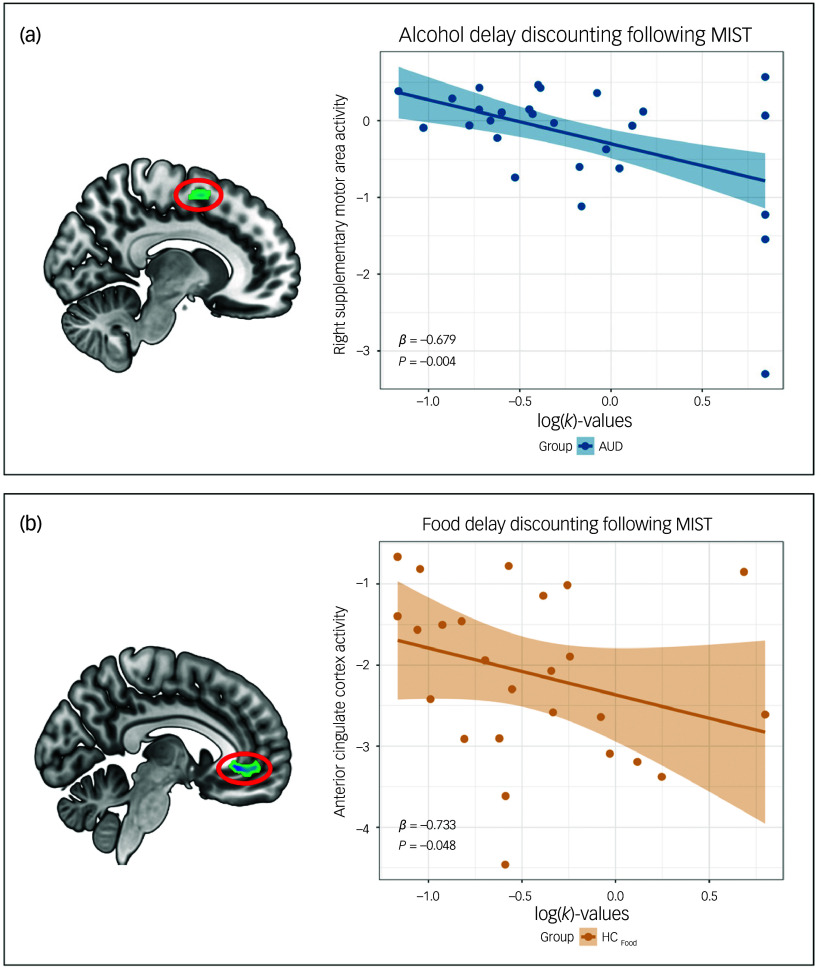
Associations between brain activity during delay discounting tasks and behavioural measures. (a) In participants with alcohol use disorder (AUD), following stress, brain activity in the right supplementary motor area during the alcohol delay discounting task (DDT) was negatively associated with log(*k*)-values (*β* = −0.679, s.e. = 0.201, *P* = 0.004); (b) in healthy controls_FOOD_, following stress, brain activity in the anterior cingulate cortex/ventromedial prefrontal cortex during the food DDT was negatively associated with log(*k*)-values (*β* = 0.733, s.e. = 0.356, *P* = 0.048). *β*, estimate; HC_FOOD_, healthy controls who performed the food DDT; MIST, Montreal Imaging Stress Task.

The results for fMRI data can be seen in Figs. 3 and 4.

#### Delay discounting of money

There were no significant differences in brain activity between the different groups (AUD versus bulimia nervosa; AUD versus healthy controls; bulimia nervosa versus healthy controls).

#### Delay discounting of food

Before MIST, participants with bulimia nervosa displayed a weaker deactivation of the left posterior insula (Montréal Neurological Institute (MNI): *x* = −47, *y* = −12, *z* = 8; *k* = 213, *t*_46_ = 4.31; *P*_FWE_ = 0.005) and right posterior insula (MNI: *x* = 36, *y* = −21, *z* = 2; *k* = 131, *t*_46_ = 4.27; *P*_FWE_ = 0.039) than healthy controls_food_. Furthermore, for bulimia nervosa, BMI was negatively associated with brain activity in the left posterior insula (*β* = −0.046, s.e. = 0.220, *P* = 0.049) and right posterior insula (*β* = −0.040, s.e. = 0.012, *P* = 0.004). In other words, weaker deactivation of the left and right posterior insulae was more pronounced in participants with a lower BMI. When comparing brain activity pre- and post-MIST, the healthy controls_food_ group displayed higher activity following MIST in the left postcentral gyrus (MNI: *x* = −26, *y* = −60, *z* = 60, *t*_48_ = 5.07, *P*_FWE_ < 0.001), right postcentral gyrus (MNI: *x* = 0, *y* = 36, *z* = 54, *t*_48_ = 4.49, *P*_FWE_ = 0.009), left SMA (MNI: *x* = −11, *y* = 7, *z* = 38, *t*_48_ = 5.15, *P*_FWE_ = 0.003) and right SMA (MNI: *x* = 17, *y* = −10, *z* = 72, *t*_48_ = 4.82, *P*_FWE_ = 0.040), but lower activity of the medial MFG/SFG (MNI: *x* = 2, *y* = 63, *z* = 18, *t*_48_ = 6.70, *P*_FWE_ < 0.001) and PCC (MNI: *x* = 4, *y* = −45, *z* = 38, *t*_48_ = 5.97, *P*_FWE_ < 0.001). Furthermore, participants with bulimia nervosa showed higher activity following MIST of the left inferior occipital, superior occipital, lingual and fusiform gyrus (MNI: *x* = −30, *y* = −66, *z* = −6, *k* = 556, *t*_48_ = 5.13, *P*_FWE_ ≤ 0.001) and right lingual and fusiform gyrus (MNI: *x* = 24, *y* = −79, *z* = −6, *k* = 137, *t*_48_ = 4.19, *P*_FWE_ = 0.021). There was no significant interaction effect between MIST task and group concerning brain activity. Comparing brain activity between groups following MIST, participants with bulimia nervosa displayed a weaker deactivation of the ACC (MNI: *x* = −2, *y* = 22, *z* = −4, *k* = 203, *t*_46_ = 4.78, *P*_FWE_ = 0.008) than healthy controls_food_. Furthermore, a lower activity of the ACC was associated with higher log(*k*)-values in healthy controls_food_ (*β* = −0.733, s.e. = 0.356, *P* = 0.048) and higher BMI (*β* = −0.083, s.e. = 0.029, *P* = 0.009) in participants with bulimia nervosa. This means that a lower activity of the ACC was related to a higher preference for more immediately available food in healthy controls_food_.

#### Delay discounting of alcohol

There were no significant differences in brain activity between participants with AUD and healthy controls_alcohol_ before MIST. Compared with pre-MIST, the AUD group displayed lower activity following MIST of the right SMA (MNI: *x* = 13, *y* = 5, *z* = 56, *k* = 123, *t*_48_ = 5.23, *P*_FWE_ = 0.007). Furthermore, lower activity of the right SMA was associated with higher log(*k*)-values following stress (*β* = −0.682, s.e. = 0.190, *P* = 0.003) in participants with AUD. This indicates that a lower activity of the right SMA was related to a higher preference for more immediately available alcohol in participants with AUD. There was no significant interaction effect between MIST task and group concerning brain activity. Comparing brain activity between groups following MIST, no significant differences were found between participants with AUD and healthy controls_alcohol_.

## Discussion

This study investigated four hypotheses. First, that those with bulimia nervosa or AUD have higher delay discounting rates for money than healthy controls. Second, that those with bulimia nervosa or AUD have higher delay discounting rates for food or alcohol than healthy controls. Third, that those with bulimia nervosa or AUD, but not healthy controls, display higher delay discounting rates for food or alcohol when stressed. Fourth, that these behavioural differences are related to brain activity changes in regions involved in the attribution and comparison of subjective value.

With regard to behaviour, this study finds no significant difference between groups concerning the delay discounting of money, food or alcohol before stress. However, it does find a nominally higher preference for more immediately available food in healthy controls following stress compared with before stress, but not in subjects with bulimia nervosa, although this did not remain after correcting for multiple testing. It also finds a significantly higher preference for more immediately available alcohol in individuals with AUD following stress compared with before stress, but not in healthy controls. Concerning brain activity, the results show that individuals with bulimia nervosa display a weaker deactivation of the left and right posterior insula while delay discounting food than healthy controls. They also show that healthy controls demonstrated a lower activity of the frontal cortex and higher activity of the motor cortex while delay discounting food following stress compared with before stress, but that those with bulimia nervosa displayed a higher activity of the occipital cortex. Furthermore, the results show that subjects with AUD display a lower activity of the right SMA while delay discounting alcohol following stress. This absence of a difference between participants and controls in the delay discounting of money, food and alcohol is unexpected, because such a difference has been found in previous studies.^7,8,10^ These negative findings could be due to our relatively small sample size; although this study meets the sample size requirements of the guidelines and includes a similar number of participants to previous studies, sample size is still limited.^7,8,10^ Future studies should therefore explore behavioural differences in food or alcohol delay discounting with a larger number of participants.

The finding that participants with AUD, but not healthy controls, prefer more immediately available alcohol following stress compared with before stress is in accordance with our hypotheses. It expands our knowledge from previous studies which show that stress can increase the value of alcohol and make individuals prefer this over other commodities such as money.^26,48^ This could be the reason why stress causes individuals to drink more alcohol and why it is an important predictor of relapse.^49,50^ Unexpectedly in this study, the higher preference for more immediately available alcohol is related to a lower activity of the right SMA, which is involved in step V (response) of the neural processing of delay discounting. Indeed, the SMA is known for its role in regulating goal-directed motor activity, but is also important for cognitive and inhibitory control.^51,52^ The lower activity of the SMA following stress could therefore reflect a loss of control over alcohol in those with AUD. Indeed, problematic drinkers have a higher tendency to lose control and act rashly when stress levels are high.^53^ Future studies should explore whether this relation between stress and alcohol delay discounting is predictive of treatment outcome, and whether it can be impacted by interventions.

The lack of a significant difference in food delay discounting between healthy controls and those with bulimia nervosa raises the question of what is signified by the weaker deactivation of the posterior insula in those with bulimia nervosa. In general, the insula is important in step II (consequences of approach) of the neural processing of delay discounting. Furthermore, previous studies show that the insula plays a role in the neural processing of food rewards, especially in the encoding of the intensity and aversity of food.^54–56^ For example, lesions of the posterior insula cause food to be perceived as less intense or unpleasant.^57,58^ Taken together, the findings of the current study suggest that those with bulimia nervosa experienced choosing the food items as either more intense or aversive. One reason why this study may have reached this conclusion is that participants were asked to select an item of food with which they could have a binge eating episode. Indeed, previous studies report that food items consumed during a binge eating episode can be ‘forbidden’ outside of such an episode.^59^ This could have made participants in the current study more inclined to restrict their food intake. If so, this would be in line with a previous study reporting that individuals with bulimia nervosa have lower delay discounting rates for food than healthy controls, meaning that they prefer the delayed food option over the immediately available one.^10^

In the current study, there was no significant change in the delay discounting of food following stress in participants with bulimia nervosa. Although previous studies have found that stress causes individuals who binge eat to eat more, most of these studies were performed in individuals with binge eating disorder who did not display compensatory behaviours such as fasting.^60^ To our knowledge, there is only one study that has investigated the impact of stress on food intake in individuals with bulimia nervosa, and it reported no effect.^61^ This suggests that the acute type of stress that is typically induced in a laboratory or neuroimaging setting does not make participants with bulimia nervosa lose control. Indeed, one previous study found that such acute stress did not reduce inhibitory control in participants with bulimia nervosa.^62^ However, studies in daily life have found that negative emotions such as stress increase before a binge eating episode in those with bulimia nervosa.^63,64^ They also found that some emotions are more related to binge eating than others (i.e. guilt versus nervousness) and that not acute stress, but the accumulation of stress, is predictive of binge eating.^65,66^ Together, these findings suggest that the relation between negative emotions and binge eating in subjects with bulimia nervosa could be dependent on the underlying emotions and their dynamics. Future neuroimaging studies should explore this by investigating the effect of various negative emotions with different designs (e.g. longer or repeated stress induction).

Although there was no significant change in food delay discounting found following stress in participants with bulimia nervosa in the current study, there was a significant change concerning brain activity. Namely, following stress, participants with bulimia nervosa showed higher activity of the occipital cortex, which is involved in step I (object representations) of the neural processing of delay discounting. This is in line with a study showing that individuals with bulimia nervosa display higher activity of the occipital cortex when viewing images of food following stress.^67^ Indeed, previous studies report that stress can lead to a higher activity of the occipital cortex and that this could be a sign of hypervigilance or amplified sensory processing.^68,69^ Therefore, these results suggest that stress makes those with bulimia nervosa process food differently, but they do not explain how. Future studies should explore how stress changes the sensory processing of food in subjects with bulimia nervosa and how this is related to certain cognitions about food.

In contrast to participants with bulimia nervosa, this study finds a nominally higher preference for immediately available food in healthy controls following stress compared with before stress. Such a difference could be due to the absence of fasting and other compensatory behaviours in healthy controls, compared with participants with bulimia nervosa. However, this should be interpreted with caution because the finding did not remain significant following correction for multiple testing. In addition, healthy controls also displayed lower activity of the PCC and medial MFG/SFG following stress compared with before stress. These regions play an important role in step III (subjective value) of the neural processing of delay discounting. A decrease in their activity could indicate that the delayed food option has less value to healthy controls following stress. If so, this could be the reason why healthy controls would be more likely to choose the immediately available food option, and would explain why lower activity in the frontal cortex following stress was related to higher log(*k*)-values.

This study has several limitations. First, the relatively small sample size could have limited the power to detect differences between participants and healthy controls, and possibly impacted the replicability of results.^70^ Second, the order of the different DDTs was not randomised within a session or separated across sessions, which could have led to practice or habituation effects. However, the decision to place the monetary DDT before the food or alcohol DDT is based on previous studies reporting that exposure to cues can impact reward processing in participants.^71^ Also, the tasks were not split across sessions to limit within-person variability. Third, as participants had not been randomised between stress and control conditions, it is possible that some effects in this study are due to fatigue or sensory-specific satiety due to the repeated use of the DDT. Fourth, most participants in this study were young Caucasian women with a short illness duration, and this limits the generalisability of the results to all those with bulimia nervosa or AUD. Future studies should aim to replicate these findings in other samples. Additionally, the young age of the participants could have influenced the absence of a difference in the delay discounting of money between participants and healthy controls, because young individuals already display higher delay discounting rates.^72^ Relatedly, a number of individuals in the healthy controls_alcohol_ group engaged in binge drinking, which could explain the similar delay discounting rates for alcohol in participants with AUD and healthy controls. Fifth, like most studies investigating the neurobiological reward system in binge eating and binge drinking, this study looked at voxel-wise brain activity;^73^ however, reward processing is more than a simple hyper- and hypoactivation of brain areas.^73^ Future studies should also either explore connectivity or perform multivariate analyses to examine neurobiological differences in delay discounting. Sixth, the decision and feedback stages were modelled with boxcar regressors, but these could also be modelled in other ways (e.g. with parametric regressors), and this may have influenced the results. Seventh, only the delay discounting of money was compared between participants with AUD and bulimia nervosa, because the commodities of the other DDTs differed between groups. Future studies could use cross-commodity DDTs to better compare both participant groups. Eighth, delay discounting was studied using *k*-values from a hyperbolic discounting model, although other measures of delay discounting exist (e.g. the area under the curve), which could have provided a different perspective on the results. Ninth, in the first-level analysis of the fMRI data, the decision and feedback stages were modelled separately and contrast images were calculated for the decision stage. This approach was chosen because combining both stages in a single boxcar regressor would be problematic, since they represent distinct cognitive processes. However, a potential limitation is the overlap of hemodynamic response functions between these stages. Additionally, alternative fMRI modelling approaches exist, which could provide additional insights in future research.

## Supporting information

Leenaerts et al. supplementary material Leenaerts et al. supplementary material

## Data Availability

The data and scripts that support the findings of this study are available upon request.

## Supplementary material

The supplementary material is available online at https://doi.org/10.1192/bjo.2025.10821
