# Supplementary material for: The effect of stress on delay discounting in female patients with early-onset bulimia nervosa and alcohol use disorder: functional magnetic resonance imaging study
Source: BJPsych Open. 2025 Sep 12;11(5):e207. doi: 10.1192/bjo.2025.10821 (PMC12451539; doi:10.1192/bjo.2025.10821)
Supplement: Leenaerts et al. supplementary material [file S2056472425108211sup001.docx]

**Supplemental Online Content**

The following items were included to give the readers more information

**eMethods 1. Recruitment**

**eMethods 2. Inclusion and exclusion criteria
eMethods 3. Study procedure**

**eFigure 1. Scanning procedure**

**eMethods 4. Food and alcohol items of the disorder-specific delay discounting task
eMethods 5. Maximally accepted delay of the delay discounting task**

**eMethods 6. Difficulty of the Montreal Imaging Stress Task**

**eMethods 7. K-values of the discounting tasks**

**eMethods 8. fMRI preprocessing.**

**eMethods 9. First-level analysis**

**eTable 1. Characteristics of the pooled healthy controls**

**eTable 2. log(k)-values of the different delay discounting tasks**

**eMethods 1. Recruitment**

Recruitment took place in Flanders, Belgium through residential and ambulatory care centers, patient groups, universities, social media, and by handing out flyers on the street.

**eMethods 2. Inclusion and exclusion criteria**

The inclusion criteria were the following: (1) female; (2) understand Dutch; (3) age ≥ 18 years; (4) BMI ≥ 18.5 kg/m2; (5) right-handed. Additional inclusion criteria for patients were: (6) meet the criteria for BN or AUD of the Diagnostic and Statistical Manual of Mental Disorders (5th ed.; APA, 2013); (7) illness duration ≤ 5 years. Participants with AUD also needed to display a pattern of repetitive BD according to the criteria of the National Institute on Alcohol Abuse and Alcoholism (i.e., drinking 4 units of alcohol within 2 hours for women) (NIAAA, 2022) . Participants were excluded for the following reasons: (1) major medical pathology; (2) chronic use of sedatives; (3) pregnancy; (4) presence of psychiatric pathology for HC or major psychiatric pathology (i.e., schizophrenia, autism spectrum disorder, bipolar disorder, substance use disorder) for patients with BN or AUD; (5) contraindication for magnetic resonance imaging (MRI); (6) known cerebral structural abnormalities. Patients could be included if they used SSRI’s, but needed to be on a stable dosage for at least four weeks.

**eMethods 3. Study procedure**

After an initial screening via telephone or email, potential participants attended an in-person assessment where a resident of psychiatry confirmed an individual’s eligibility to participate. Additionally, the participants had their weight and height measured with a calibrated scale and stadiometer, completed clinical interviews and questionnaires, and were scheduled for the MRI scan session. The participants controlled their own diet in the days leading up to the scan.

**eFigure 1. Scanning procedure**


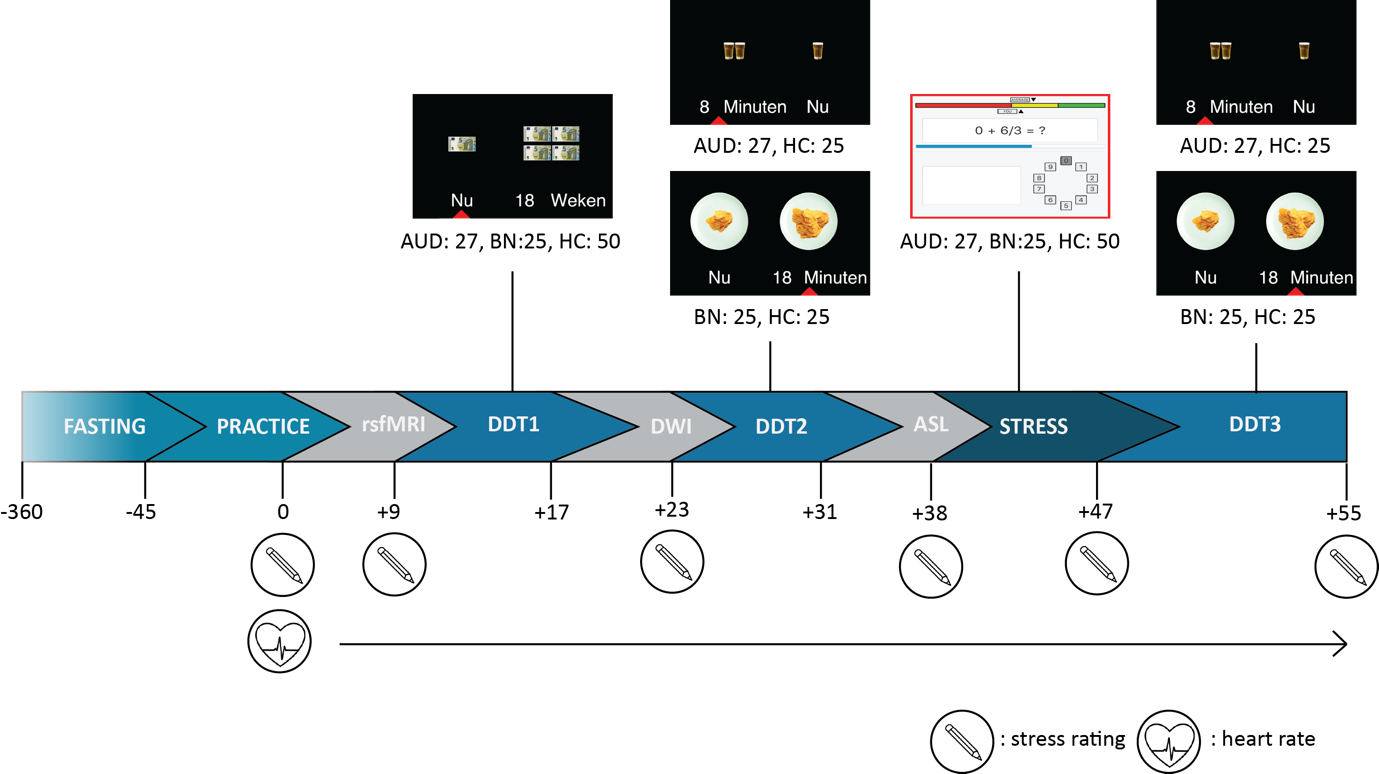
All participants underwent an initial resting-state fMRI (rsfMRI) scan, during which they were instructed to keep their eyes closed but remain awake. Following this, they completed a monetary delay discounting task (DDT) and then a food or alcohol delay discounting task DDT. Stress was then induced using the Montreal Imaging Stress Task (MIST), and a T1-weighted structural image was acquired during this task. After the MIST, participants repeated the food or alcohol DDT. The DDTs were spaced apart by the acquisition of arterial spin labeling (ASL) and diffusion-weighted imaging (DWI) data. Throughout the scanning process, participants reported their stress levels, and their heart rate was monitored using a photoplethysmography sensor. Abbreviations: AUD, alcohol use disorder; ASL, arterial spin labeling; BN, bulimia nervosa; DDT, delay discounting task; DWI, diffusion-weighted imaging; HC, healthy control, rsfMRI, resting-state functional magnetic resonance imaging.

**eMethods 4. Items of the food and alcohol delay discounting task**

In the practice session, patients with BN or AUD were asked to select an item of food or alcohol they could have a BE or BD episode with and which they would like to eat or drink right now. The HC were asked to select an item of food or alcohol they could eat a lot of and which they would like to eat or drink right now. This item was then used in the food or alcohol delay discounting task (DDT).

Food:

- Pizza
- Donuts
- Chocolate
- Cookies
- Cake
- Pancakes
- Ice cream
- Cupcakes
- Potato chips
- Candy

Alcohol:

- Beer
- White wine
- Red wine
- Liquor
- Cocktail

**eMethods 5. Maximally accepted delay of the delay discounting tasks**

All participants completed a practice version of the monetary DDT and the food or alcohol DDT in the practice session. This version of the DDT was similar to the ones used in the scanner, except for the delays and the number of trials. The practice version of the DDT used a fixed set of 22 delays (1, 2, 3, 4, 5, 6, 7, 8, 9, 10, 12, 14, 16, 18, 20, 30, 40, 50, 60, 75, 90, 120) which were paired with each delayed reward (2-5x the immediate reward), resulting in a total of 88 trials per DDT. The delays for the monetary DDT were expressed in weeks, whereas the delays for the food or alcohol DDT were expressed in minutes. The maximally accepted delay for the DDTs was defined as the largest delay at which a participant a participant still chose the delayed reward. This is because as the delay increases, the value of a delayed reward decreases, until at a certain point the immediate reward is always chosen over the delayed reward (Odum, 2011). Afterwards, the deciles of the maximally accepted delay plus ten percent were used as the delays for the DDTs in the scanner. For example, if a participants maximally accepted delay was 20, then this was multiplied by 1.1, which results in in 22, which was then split into its deciles (2.2,4.4,6.6,8.8,11,13.2,15.4,17.8,20,22.2). This resulted in a DDT in the scanner that was similar those the DDT of the practice session, but without the choices where participants always chose the immediate option.

**eMethods 6. Difficulty of the Montreal Imaging Stress Task**

During the practice session, participants performed a control version of the MIST with as much time as necessary, no fictive score to compete with, and no negative feedback. They performed this version at four different difficulty levels for three minutes each. The difficulty level for the experimental version in the scanner was determined as the highest level where at least 80% of the responses were within 5 seconds (Wheelock et al., 2016).

**eMethods 7. K-values of the discounting tasks**

As in previous work, A k-value was estimated for every DDT by fitting the choice data to a hyperbolic discounting model (Kable & Glimcher, 2007; Weygandt et al., 2019). In this model, the subjective value (SV) of the delayed reward was defined by a hyperbolic function of the objective value (M), the delay (D) and the k-value. For this study, the objective value of the delayed reward corresponded to the multiple of the immediate reward (2-5). The hyperbolic function was described by the following formula:


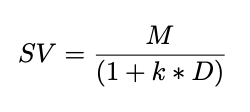


From a set of systematically varied k-values, a k-value was selected which minimized the sum of squared differences between the true SV of the delayed rewards based on the DDT (i.e., 1 when the delayed reward was selected and 0 when the delayed reward was not selected) and the predicted SV of the delayed rewards of the formula (Weygandt et al., 2019).

**eMethods 8. fMRI preprocessing.**

All fMRI data were preprocessed with fmriprep, version 21.0.1. (Esteban et al., 2019). For each of the BOLD runs found per subject (across all tasks and sessions), the following preprocessing was performed. First, a reference volume and its skull-stripped version were generated using a custom methodology of fMRIPrep. Head-motion parameters with respect to the BOLD reference (transformation matrices, and six corresponding rotation and translation parameters) are estimated before any spatiotemporal filtering using mcflirt (FSL 6.0.5.1:57b01774, Jenkinson et al. 2002). BOLD runs were slice-time corrected to 0.409s (0.5 of slice acquisition range 0s-0.818s) using 3dTshift from AFNI (Cox and Hyde 1997, RRID:SCR_005927). The BOLD time-series (including slice-timing correction when applied) were resampled onto their original, native space by applying the transforms to correct for head-motion. These resampled BOLD time-series will be referred to as preprocessed BOLD in original space, or just preprocessed BOLD. The BOLD reference was then co-registered to the T1w reference using mri_coreg (FreeSurfer) followed by flirt (FSL 6.0.5.1:57b01774, Jenkinson and Smith 2001) with the boundary-based registration (Greve and Fischl 2009) cost-function. Co-registration was configured with six degrees of freedom. Several confounding time-series were calculated based on the preprocessed BOLD: framewise displacement (FD), DVARS and three region-wise global signals. FD was computed using two formulations following Power (absolute sum of relative motions, Power et al. (2014)) and Jenkinson (relative root mean square displacement between affines, Jenkinson et al. (2002)). FD and DVARS are calculated for each functional run, both using their implementations in Nipype (following the definitions by Power et al. 2014). The three global signals are extracted within the CSF, the WM, and the whole-brain masks. Additionally, a set of physiological regressors were extracted to allow for component-based noise correction (CompCor, Behzadi et al. 2007). Principal components are estimated after high-pass filtering the preprocessed BOLD time-series (using a discrete cosine filter with 128s cut-off) for the two CompCor variants: temporal (tCompCor) and anatomical (aCompCor). tCompCor components are then calculated from the top 2% variable voxels within the brain mask. For aCompCor, three probabilistic masks (CSF, WM and combined CSF+WM) are generated in anatomical space. The implementation differs from that of Behzadi et al. in that instead of eroding the masks by 2 pixels on BOLD space, the aCompCor masks are subtracted a mask of pixels that likely contain a volume fraction of GM. This mask is obtained by thresholding the corresponding partial volume map at 0.05, and it ensures components are not extracted from voxels containing a minimal fraction of GM. Finally, these masks are resampled into BOLD space and binarized by thresholding at 0.99 (as in the original implementation). Components are also calculated separately within the WM and CSF masks. For each CompCor decomposition, the k components with the largest singular values are retained, such that the retained components’ time series are sufficient to explain 50 percent of variance across the nuisance mask (CSF, WM, combined, or temporal). The remaining components are dropped from consideration. The head-motion estimates calculated in the correction step were also placed within the corresponding confounds file. The confound time series derived from head motion estimates and global signals were expanded with the inclusion of temporal derivatives and quadratic terms for each (Satterthwaite et al. 2013). Frames that exceeded a threshold of 0.5 mm FD or 1.5 standardised DVARS were annotated as motion outliers. The BOLD time-series were resampled into several standard spaces, correspondingly generating the following spatially-normalized, preprocessed BOLD runs: MNI152NLin6Asym, MNI152NLin2009cAsym. First, a reference volume and its skull-stripped version were generated using a custom methodology of fMRIPrep. Automatic removal of motion artifacts using independent component analysis (ICA-AROMA, Pruim et al. 2015) was performed on the preprocessed BOLD on MNI space time-series after removal of non-steady state volumes and spatial smoothing with an isotropic, Gaussian kernel of 6mm FWHM (full-width half-maximum). Corresponding “non-aggresively” denoised runs were produced after such smoothing. Additionally, the “aggressive” noise-regressors were collected and placed in the corresponding confounds file. All resamplings can be performed with a single interpolation step by composing all the pertinent transformations (i.e. head-motion transform matrices, susceptibility distortion correction when available, and co-registrations to anatomical and output spaces). Gridded (volumetric) resamplings were performed using antsApplyTransforms (ANTs), configured with Lanczos interpolation to minimize the smoothing effects of other kernels (Lanczos 1964). Non-gridded (surface) resamplings were performed using mri_vol2surf (FreeSurfer). Many internal operations of fMRIPrep use Nilearn 0.8.1 (Abraham et al. 2014, RRID:SCR_001362), mostly within the functional processing workflow. For more details of the pipeline, see the section corresponding to workflows in fMRIPrep’s documentation. Finally, the fMRI data were smoothed with an 8 mm full width at half maximum (FWHM) Gaussian kernel in SPM12.

**eMethods 9. First-level analysis**

The first-level analysis used a general linear model with boxcar regressors which separately modeled the decision and feedback stages. These boxcar regressors were convolved with the canonical hemodynamic response function without any spatial or temporal derivatives. Additionally, 3 rotation, 3 translation, 6 derivatives, 5 wCompCor, 5 cCompCor and 5 cosine nuisance regressors were added to the general linear model. Previous research has shown that the traditional rotation and translation parameters, even with derivatives, are not sufficient to remove head motion artefacts (Parkes et al., 2018). Research has also shown that the inclusion of CompCor regressors together with the global signal can significantly reduce the impact of head motion on the fMRI signal (Muschelli et al., 2014). Therefore, it was chosen to include both the head motion parameters as well as the CompCor variables as nuisance regressors. The inclusion of 5 cosine regressors was advised by fmriprep as the program performs a high-pass filter before running its CompCor algorithm (Esteban et al., 2019). Because of the inclusion of these regressors, no additional high-pass filter was applied in SPM12. Temporal correlations were modeled with a AR(1) structure of the errors. From this first-level analysis, contrast images were calculated for the decision stages.

|  |  |
| --- | --- |
| **eTable 1. Characteristics of the pooled healthy controls** | |
|  | HC (n=50) |
|  | Mean (SD) |
| Age | 21.6 (2.5) |
| BMI | 22.3 (2.2) |
| Education (years) | 15.1 (1.7) |
| AUDIT | 3.5 (2.1) |
| EDE-Q  Restraint  Shape Concern  Weight Concern  Eating Concern  Total | 0.4 (0.7)  1.0 (1.0)  0.8 (1.0)  0.2 (0.4)  0.6 (0.7) |
| DASS  Total  Anxiety  Depression  Stress | 12.1 (10.1)  2.6 (3.3)  2.9 (3.2)  6.6 (4.9) |
|  | n (%) |
| Binge drinking frequency  Never  Annually  Semi-annually  Three-monthly  Monthly  Biweekly  Weekly  >Weekly | 26 (52%)  3 (6%)  6 (12%)  9 (18%)  5 (10%)  1 (2%)  0 (0%)  0 (0%) |
| Ethnicity  Caucasian  Asian  Mixed | 48 (96%)  1 (2%)  1 (2%) |
| Contraceptive use | 46 (92%) |

| **eTable 2. log(k)-values of the different delay discounting tasks** | | | | |
| --- | --- | --- | --- | --- |
| Variable | AUD (n=27) | HC (n=50) | | BN (n=25) |
| DD money | -0.46 (0.53) | -0.58 (0.44) | | -0.40 (0.55) |
| Variable | AUD (n=27) | HC_alcohol_ (n=25) | HC_food_ (n=25) | BN (n=25) |
| DD food/alcohol before MIST | -0.28 (0.60) | 0.07 (0.67) | -0.54 (0.41) | -0.62 (0.34) |
| DD food/alcohol after MIST | -0.21 (0.61) | 0.07 (0.68) | -0.46 (0.53) | -0.56 (0.42) |
| Variables are represented as mean (standard deviation). Abbreviations: AUD, alcohol use disorder; BN, bulimia nervosa; DD, delay discounting; HC, healthy control; HC_alcohol_, healthy controls who performed the alcohol delay discounting task; HCfood; MIST, Montreal imaging stress task. | | | | |
